# Supplementary figures and images for: Occasional hybridization between a native and invasive Senecio species in Australia is unlikely to contribute to invasive success
Source: PeerJ. 2017 Aug 15;5:e3630. doi: 10.7717/peerj.3630 (PMC5562138; doi:10.7717/peerj.3630)

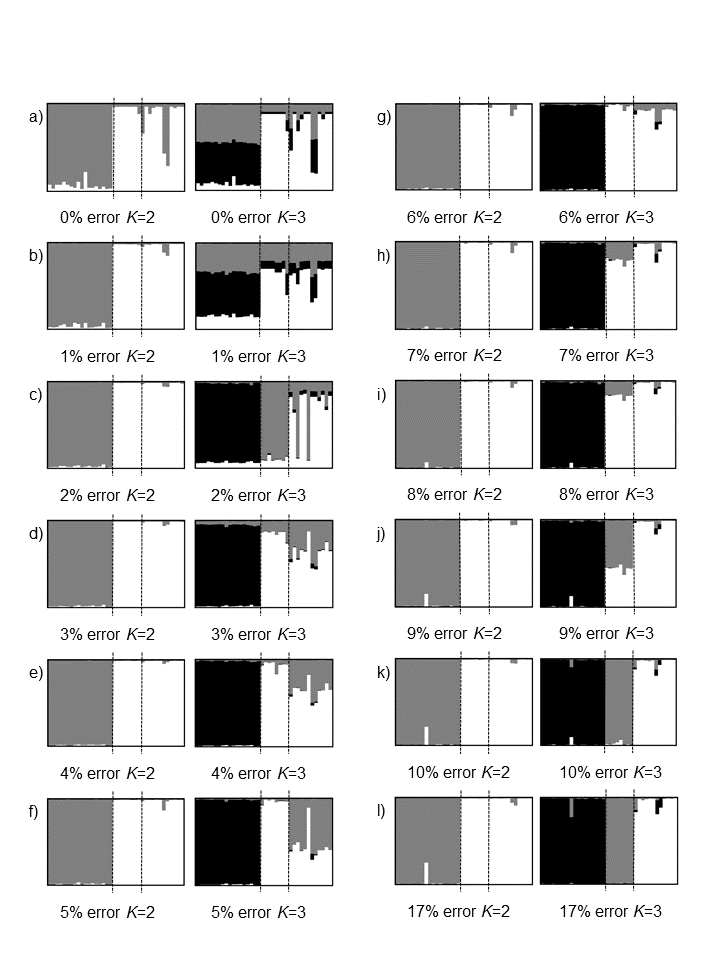

Supplement: Figure S1 — Only allopatric populations of Senecio madagascariensis and Senecio pinnatifolius were used to avoid the confounding effect that detection of hybrids might have on the output. S.madagascariensis is shown on the left side of the plots, S. pinnatifolius ‘dune variant’ on the right. Results are shown for K = 2 (equating to two species) and K = 3 (number of different plates the samples were run on). The dotted lines represent the plate boundaries. The final dataset chosen is shown in (g) where both species are clearly define and at K = 3 there are no obvious plate effects. (a) 0% error, 33 loci; (b) 1% error, 56 loci; (c) 2% error, 79 loci; (d) 3% error, 96 loci; (e) 4% error, 112 loci; (f) 5% error, 128 loci; (g) 6% error, 141 loci; (h) 7% error, 154 loci; (i) 8% error, 165 loci; (j) 9% error, 175 loci; (k) 10% error, 184 loci; (l) 17% error, 233 loci. [file peerj-05-3630-s002.png]

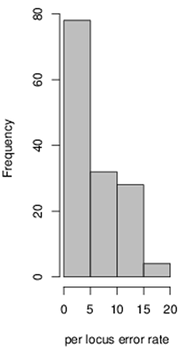

Supplement: Figure S2 — Frequency histogram of locus specific error rates in the final AFLP dataset with an overall mean error rate of 6% across 141 loci. [file peerj-05-3630-s003.png]
